# Prevalence and network structure of depression, insomnia and suicidality among mental health professionals who recovered from COVID-19: a national survey in China

**DOI:** 10.1038/s41398-024-02918-8

**Published:** 2024-05-30

**Authors:** He-Li Sun, Pan Chen, Wei Bai, Ling Zhang, Yuan Feng, Zhaohui Su, Teris Cheung, Gabor S. Ungvari, Xi-Ling Cui, Chee H. Ng, Feng-Rong An, Yu-Tao Xiang

**Affiliations:** 1 Unit of Psychiatry, Department of Public Health and Medicinal Administration, & Institute of Translational Medicine, Faculty of Health Sciences, University of Macau, Macao SAR, China; 2 Centre for Cognitive and Brain Sciences, University of Macau, Macao SAR, China; 3 Department of Epidemiology and Biostatistics, School of Public Health, Jilin University, Changchun, China; 4 Beijing Key Laboratory of Mental Disorders, National Clinical Research Center for Mental Disorders & National Center for Mental Disorders, Beijing Anding Hospital; Advanced Innovation Center for Human Brain Protection, Capital Medical University, Beijing, China; 5 School of Public Health, Southeast University, Nanjing, China; 6 School of Nursing, Hong Kong Polytechnic University, Hong Kong SAR, China; 7 Section of Psychiatry, University of Notre Dame Australia, Fremantle, WA Australia; 8 Division of Psychiatry, School of Medicine, University of Western Australia, Perth, WA Australia; 9 Department of Business Administration, Hong Kong Shue Yan University, Hong Kong, Hong Kong SAR China; 10 Department of Psychiatry, The Melbourne Clinic and St Vincent’s Hospital, University of Melbourne, Richmond, VIC Australia

**Keywords:** Diseases, Depression

## Abstract

Psychiatric syndromes are common following recovery from Coronavirus Disease 2019 (COVID-19) infection. This study investigated the prevalence and the network structure of depression, insomnia, and suicidality among mental health professionals (MHPs) who recovered from COVID-19. Depression and insomnia were assessed with the Patient Health Questionnaire (PHQ-9) and Insomnia Severity Index questionnaire (ISI7) respectively. Suicidality items comprising suicidal ideation, suicidal plan and suicidal attempt were evaluated with binary response (no/yes) items. Network analyses with Ising model were conducted to identify the central symptoms of the network and their links to suicidality. A total of 9858 COVID-19 survivors were enrolled in a survey of MHPs. The prevalence of depression and insomnia were 47.10% (95% confidence interval (CI) = 46.09–48.06%) and 36.2% (95%CI = 35.35–37.21%), respectively, while the overall prevalence of suicidality was 7.8% (95%CI = 7.31–8.37%). The key central nodes included “Distress caused by the sleep difficulties” (ISI7) (EI = 1.34), “Interference with daytime functioning” (ISI5) (EI = 1.08), and “Sleep dissatisfaction” (ISI4) (EI = 0.74). “Fatigue” (PHQ4) (Bridge EI = 1.98), “Distress caused by sleep difficulties” (ISI7) (Bridge EI = 1.71), and “Motor Disturbances” (PHQ8) (Bridge EI = 1.67) were important bridge symptoms. The flow network indicated that the edge between the nodes of “Suicidality” (SU) and “Guilt” (PHQ6) showed the strongest connection (Edge Weight= 1.17, followed by “Suicidality” (SU) - “Sad mood” (PHQ2) (Edge Weight = 0.68)). The network analysis results suggest that insomnia symptoms play a critical role in the activation of the insomnia-depression-suicidality network model of COVID-19 survivors, while suicidality is more susceptible to the influence of depressive symptoms. These findings may have implications for developing prevention and intervention strategies for mental health conditions following recovery from COVID-19.

## Introduction

Since December 2022, there had been a surge in Coronavirus Disease 2019 (COVID-19) cases in China following a swift relaxation of its stringent zero-COVID restrictions [1–3]. According to a key source at the Center of Disease Control and Prevention, approximately 80% of the population in China could have been infected with COVID-19 by the end of January 2023 [4]. The majority of the COVID-19 patients experienced minimal or mild symptoms, with only a few cases of severe respiratory distress or failure requiring ICU care during that time [5]. However, a substantial number of COVID-19 patients might have had post-COVID-19 related symptoms, which could persist for at least 2 months following the acute phase of infection [6, 7].

The post-COVID-19 condition, which is also known as long COVID-19, can include fatigue, pain, dizziness, sore throat, and so on [8]. Apart from somatic symptoms, psychiatric symptoms are also common in patients after COVID-19 infection [9]. A study conducted in the early stage of the COVID-19 pandemic found that 22.5% of adult patients with COVID-19 had neuropsychiatric comorbidities, particularly depression and sleep problems [10]. Studies also found that depression was one of the most common psychiatric symptoms experienced by COVID-19 survivors, with a prevalence ranging from 4 to 31% at 1 month following infection [11]. Additionally, a prospective study of COVID-19 patients found that 22.34% of participants had suffered insomnia after 1 year follow-up [12]. Previous research have also indicated that insomnia is frequently comorbid with depression [13], and those with psychiatric problems are prone to suicidal thoughts or behaviors [14–17]. Therefore, it is important to further understand the associations between depression, insomnia, and suicidal symptoms among COVID-19 survivors.

Network analysis is an innovative approach to examine how symptoms are interconnected [18]. In contrast to traditional latent variable approaches that regard symptoms as independent and equally important indicators of a syndrome, the network approach views each symptom as a contributing factor that can directly influence or be influenced by other symptoms even if the disorder is not present [19]. In a network model, individual symptoms are represented as nodes, while the connections between them are represented as edges [20]. Moreover, it offers the opportunity to identify critical nodes and relevant influential connections within a network model that could serve as potential targets for interventions to address the specific syndrome [19].

In recent years, network analysis has been widely used to assess the interaction between depression and insomnia at the symptom level during the COVID-19 pandemic in the general population and other subpopulations, such as psychiatric professionals [21–23]. To the best of our knowledge, no previous network analysis studies had examined the network structure of depression, insomnia, and suicidality among mental health professionals (MPHs) who had recovered from COVID-19.

Further, in order to understand the inter-relationships of psychiatric symptoms, especially those related to suicidality in COVID-19 survivors, this study examined the prevalence of depression, insomnia and suicidality in MPHs who had recovered from COVID-19 infection, and also explored the inter-relationships between depressive and insomnia symptoms, and suicidality using network analysis. Considering the mental health challenges in MHPs due to both infection and work burden [24, 25], we hypothesize that depressive and insomnia symptoms are common in MPHs who had recovered from COVID-19 inflection.

## Methods

### Study design and participants

This was a cross-sectional, national survey carried out by panel members of the Chinese Society of Psychiatry and the Psychiatry Branch, Chinese Nursing Association between January 22, 2023 and February 10, 2023 (i.e., immediately after the end of China’s Dynamic Zero-COVID Policy) using a convenience sampling approach. To avoid the potential risk of infection during the COVID-19 pandemic, an online survey with WeChat-based Questionnaire was performed. As all mental health workers in China needed to report their health status each day via WeChat during the pandemic, all mental health workers were presumably WeChat users. A Quick Response Code (QR code) linked to the study invitation and questionnaire generated by the WeChat-based Questionnaire Star program was distributed to all public psychiatric hospitals nationwide. In this study, psychiatrists, nurses, and technicians who (1) were 18 years old and above, (2) worked in psychiatric hospitals or psychiatric departments of general hospitals during the COVID-19 pandemic and during the study period, (3) had recovered from COVID-19 (i.e., COVID-19 test result was negative when they participated in this survey), and (4) were able to understand Chinese and provide written informed consent, were included. The study protocol was approved by the Ethics Committee of the Beijing Anding Hospital, China.

### Measurement

Basic demographic and clinical characteristics were collected, including age, gender, marital status, and education level. Participants who had recovered from COVID-19 were defined by asking the question “Since 1 December 2022, have you ever been infected with COVID-19?”, with a score of 0 for “never been infected with COVID-19”, 1 for “Infected but not hospitalized”, and 2 and “Infected and hospitalized”. Participants who selected ‘1’ or ‘2’, with negative COVID-19 test when they participated in this survey, were considered as “participants who had recovered from COVID-19 infection” (i.e., COVID-19 survivors).

Depressive symptoms in the past week were assessed with the validated Chinese version of the nine-items Patient Health Questionnaire (PHQ-9) [26, 27]; The PHQ-9 has been widely used with good validity and reliability in health workers [28]. Each item was scored based on “0” (not at all) to “1” (nearly every day), with the sum score ranging from 0 to 27, and a higher score reflecting more severe depressive symptoms. As recommended by previous studies [29, 30], participants with a total score ≥5 were considered as “having depressive symptoms” (hereafter with depression).

Insomnia symptoms in the past week were measured using the validated Chinese version of the seven-item Insomnia Severity Index questionnaire (ISI7) [31, 32]. Each item is scored based on a 5-point Likert scale, from “0” (no problem) to “4” (very severe problem). The total score ranged from 0 to 28, and a higher score indicating more severe insomnia symptoms. Those with a cut-off value ≥8 were considered as “having insomnia symptoms (hereafter with insomnia) [33].

Three suicide-related items (e.g., suicidal ideation, suicidal plan, and suicidal attempt) during the past week were assessed using the following standard questions [34]: “Have you had thoughts that you would be better off dead?”, “Have you made a plan for suicide?”, and “Have you attempted suicide?”, respectively. Each question was coded by “0” (no) and “1” (yes). Participants who answered “yes” to any of the three questions were defined as having “suicidality” [29].

### Data analysis

#### Network estimation

Redundant identification was the prerequisite of constructing the network model; a high similarity between two nodes was considered as redundancy [35], specifically, when two nodes having more than 75% similar correlations with other nodes in the network at statistical level (*p* = 0.05). Based on previous research [36–38], the goldbricker function was used to screen the redundant pairs. If any redundant pairs were identified, one of the overlapped symptoms was removed before estimating the network model [35]. Additionally, the normal distribution of the data was examined. As the data exhibited skewness, the data were transformed into binary variables following previous research [39].

The network model of depression, insomnia, and suicidality was built with the Ising model, which was used for binary data [18, 19]. Responses to the PHQ-9 and ISI7 items were recorded as “1” (raw scores larger than 0) or “0” (raw scores of 0), representing with or without the symptoms, respectively. In addition, the eLasso procedure was applied to increase the specificity of edges within the network. In the network theory, symptoms were regarded as nodes, associations between symptoms were regarded as edges, while symptoms within the same psychiatric syndrome were viewed as one cluster. This network model included three clusters (e.g., depression, insomnia, and suicidality) [19, 40]. In addition, the network in a flow diagram was set up to examine the inter-relationships of suicidality with different depressive and insomnia symptoms in the network. Network estimation and redundancy were assessed using the “*networktools*” packages [41].

#### Node centrality and predictivity

We used the expected influence (EI) to identify the most central symptoms of the depression, insomnia, and suicidality network model, which might reflect both positive and negative associations with surrounding nodes, thus indicating how strongly a node was directly connected to others in the network [42]. EI was the most commonly used centrality index and was more stable compared with other traditional centrality statistics, such as strength, betweenness, and closeness [43]. We also calculated the bridge EI, which could examine symptoms that strongly connect different symptom cluster and indicate core symptoms that activate the co-occurrence of different clusters. The predictability of nodes was assessed with CCmarg value [44]. Node centrality of EI was calculated using “qgraph” package [45], while CCmarg values were evaluated using “mgm” package [46].

#### Network stability and accuracy

The network stability was evaluated by the correlation stability coefficient (CS-coefficient) through a case-drop bootstrapping procedure [47]; CS-coefficient larger than 0.25 indicated nodes had moderate stability, while values greater than 0.5 indicated strong stability [48]. The network accuracy was assessed by bootstrapped 95% confidence intervals (CIs) through a non-parametric bootstrapping procedure [47], with a narrower CI having a more accurate network. Finally, we performed a bootstrapped difference test between the EI values and between weights of edge to identify whether the nodes and edges were different from each other [47]. Node stability and accuracy were examined using “bootnet” packages [18]. Data analyses were performed using the R program [49].

## Results

### Participants information

Altogether, 11760 mental health workers were invited to participate in this survey, of whom, 9858 met the inclusion criteria and were included in analyses. Basic demographic and clinical characteristics are shown in Table 1. The mean age of participants was 38.24 years (standard deviation = 8.302), and the majority of the participants were married (73.1%). The prevalence rate of depression and insomnia were 47.10% (95% confidence interval (CI) = 46.09–48.06%) and 36.2% (95%CI = 35.35–37.21%), respectively. The overall prevalence of suicidality was 7.8% (95%CI = 7.31–8.37%), while the prevalence of SI, SP, and SA were 6.5% (95%CI = 6.00–6.97%), 2.7% (95%CI = 2.40–3.04%), and 3.0% (95%CI = 2.64–3.31%), respectively.

**Table 1:** Basic demographic and clinical characteristics (*N* = 9858).

Variables	*N*	%
Male	1750	17.8
Married	7209	73.1
University and above	9346	94.8
Hospitalization	163	1.7
Depression (PHQ-9 ≥5)	4640	47.1
Insomnia (ISI ≥8)	3575	36.2
Comorbid depression and insomnia (PHQ-9 ≥5) & (ISI ≥8)	2947	30.2
Any type of suicidality	771	7.8
Suicidal ideation	638	6.5
Suicidal plan	266	2.7
Suicidal attempt	292	3.0
	**Mean**	**SD**
Age (years)	34.82	8.302
PHQ-9 total	5.39	5.413
ISI7 total	6.18	5.462

*Notes: PHQ-9* patient health questionnaire; *ISI* insomnia severity index.

### Network structure and centrality

**Fig. 1:**
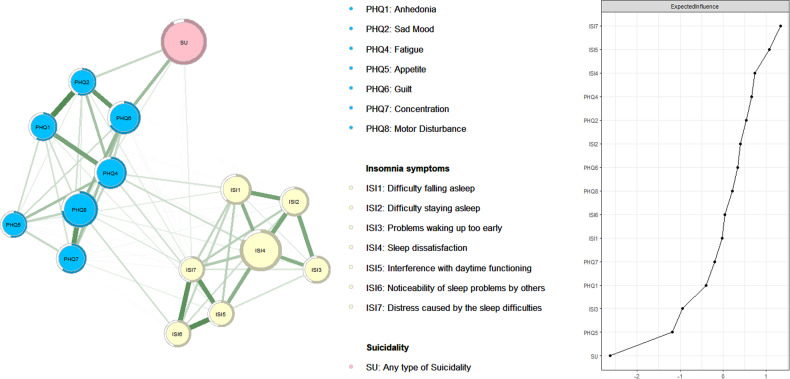
The network structure of depression and insomnia with suicidality in MPHs who recovered from COVID-19. Left panel: Network structure of depression, insomnia and suicidality; Right panel: Centrality index of EI for each node.

Figure 1 presents the network structure of the depression, insomnia, and suicidality model in MHPs who recovered from COVID-19 with a total of 15 nodes. The mean predictability of the nodes was 0.878, suggesting that 87.8% of nodes could be predicted by their neighboring nodes in the model. The top three central nodes that activated the whole network (Z score of expected influence) were “Distress caused by the sleep difficulties” (ISI7) (EI = 1.34), “Interference with daytime functioning” (ISI5) (EI = 1.08), and “Sleep dissatisfaction” (ISI4) (EI = 0.74). The EI values are presented in Table S1.

**Fig. 2:**
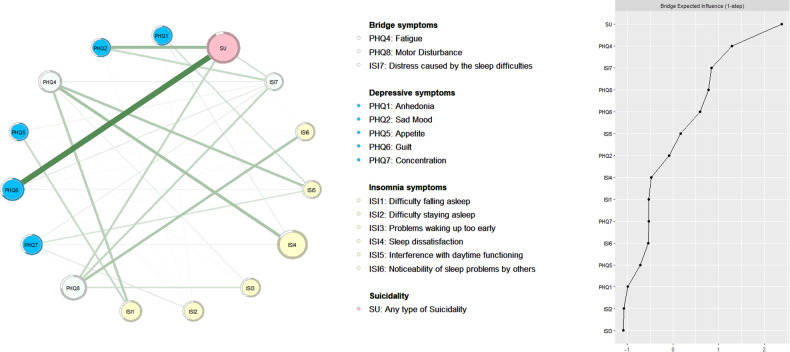
The bridge network structure of depression, insomnia, and suicidality in MPHs who recovered from COVID-19. Left panel: Network structure of depression, insomnia and suicidality; Right panel: Bridge centrality index of BEI for each node.

Figure 2 shows the edges across different clusters in the network and the rank of bridge EI values (1-step Bridge Expected Influence). Expected “Suicidality” (SU) (Bridge EI = 2.66) (which was regarded as a bridge symptom since it was a cluster composed of a single node), “Fatigue” (PHQ4) (Bridge EI = 1.98), “Distress caused by the sleep difficulties” (ISI7) (Bridge EI = 1.71) and “Motor Disturbances” (PHQ8) (Bridge EI = 1.67) were important bridge symptoms that linked the whole network.

**Fig. 3:**
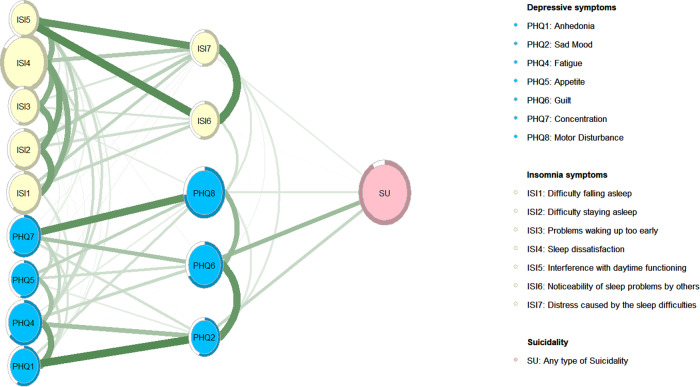
Flow network of depression and insomnia with suicidality in MPHs who recovered from COVID-19. Green edges represent positive partial correlations.

Figure 3 shows the flow diagram of the network model, which indicates in descending order that “Guilt” (PHQ6) (Edge weight = 1.17), and “Sad Mood” (PHQ2) (Edge weight = 0.67) in the depression cluster were directly associated with suicidality with strong weights, while the association between guilt and suicidality was the strongest. In addition, “Distress caused by the sleep difficulties” (ISI7) (Edge weight = 0.31) in the insomnia cluster were directly associated with suicidality with weak weights. The edge-weighted values are presented in Table S2.

### Network stability and accuracy

**Fig. 4:**
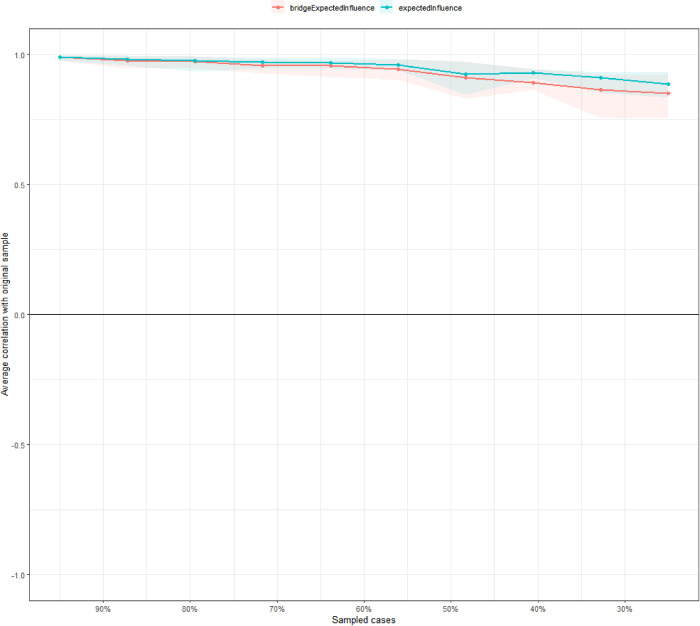
Centrality and bridge centrality stability tests. Average correlation between the centrality indices with cases dropped and the original sample. The lines indicate the means and the areas indicate the range from 10% to 90% quantile.

In terms of network stability shown in Fig. 4, an excellent level of stability was observed in both EI and bridge EI, with CS-coefficient being 0.75 and 0.67, respectively. Figures S1 and S2 display the Bootstrapped 95% CIs, indicating that the estimated EIs and bridge EI were reliable and stable. The bootstrap difference test in Figs. S3, S4 indicates that most edges and EIs were significantly different from others.

## Discussion

To the best of our knowledge, this was the first study globally that examined the prevalence and network structure of depression, insomnia, and suicidality among MHPs who recovered from COVID-19 infection. Our findings found that the three most central symptoms in the network were in the insomnia cluster, whereas the three strongest associations with suicidality were observed in the depressive cluster.

The prevalence of depression and insomnia was high in MHPs who recovered from COVID-19, which is consistent with previous meta-analyses that found that the prevalence of depression among doctors during the COVID-19 pandemic was 20.5% [50], while the pool prevalence of depression in COVID-19 infected patients was 45% [51]. As for insomnia, a meta-analysis conducted during the COVID-19 pandemic showed that the prevalence of insomnia in healthcare workers and COVID-19 patients were 46.4% and 48.7%, respectively, which were roughly twice as high as that in the general population (26.0%) [52]. These findings appear to be consistent with the hypothesis of this study.

It was noted that the rapid lifting of the stringent zero-COVID-19 restrictions had put an unprecedented strain on China’s healthcare system [53]. COVID-19-infected MPHs faced dual pressure from both their work and illness recovery, thus experiencing a greater likelihood of depression or insomnia than the general population. Further, in that period, most MHPs were re-deployed to care for COVID-19 patients and faced with higher work pressure and longer work shifts, which could increase the risk of insomnia [54]. Moreover, during their own recovery from COVID-19, MHPs were often directly involved in treating COVID-19 patients, which increased their risk for secondary infection as well as depressive and insomnia symptoms [55].

“Distress caused by sleep difficulties” (ISI7), which reflects the level of worry caused by sleep problems, was the most central and important bridge symptom among MHPs who had recovered from COVID-19 inflection, thus suggesting it could more readily influence other symptoms within the depression-insomnia-suicidality network model [48]. As a fundamental aspect of anxiety [56], worry also plays a crucial role in maintaining insomnia and predicting depression [57]. This is consistent with the findings of another network analysis study in a community-dwelling population during the COVID-19 pandemic, in which anxiety symptoms were the most important bridge symptoms linking depression and insomnia [22]. One potential explanation is that distress caused by sleep problems is usually accompanied by a range of negative emotions in patients who recovered from an infection [58]. MHPs who had recovered from COVID-19 might worry about various issues such as patients’ safety, shortage of medicine, and the risk of infecting their families and friends, all of which could exacerbate depression [59]. Moreover, there was a direct connection between “Distress caused by sleep difficulties” (ISI7) and “Suicidality” (SU) in the flow network, indicating that interventions aimed at addressing worry and distress arising from sleep difficulties could be useful in reducing both depressive symptoms and suicidality in this subpopulation. For instance, a widely used approach is cognitive behavior therapy, which has good evidence in treating insomnia related worries [60].

Another insomnia symptom “interference with daytime functioning” (ISI5) (i.e., trouble with concentration, mood disturbances, or decreased ability to perform daily activities) was identified as a core symptom in the network model. For patients who recovered from COVID-19, problems with memory, concentration, or sleep were among the most common post-COVID-19 symptoms, even after an extended time following infection [8, 61]. These impairments could considerably affect daily work [8], especially for MPHs who were required to return to work immediately despite having post-COVID-19 conditions, which consequently impeded their ability to work effectively [62].

“Sleep dissatisfaction” (ISI4), which refers to the subjective feelings of sleep quality, also had a central role in activating and maintaining the network model of depression-insomnia-suicidality in COVID-19 survivors. This is in line with previous studies which found that sleep dissatisfaction was a common feature in patients with depression [63, 64] and those who were dissatisfied with sleep were at higher risk of developing mental disorders than those who were not [65]. Moreover, self-reported sleep dissatisfaction were found to be significantly associated with suicidal ideation in the past year [66].

Depressive symptom “Fatigue” (PHQ4) was one of the top bridge symptoms within the depression-insomnia-suicidality network model. Previous research found that approximately one-third of people experienced fatigue after being diagnosed with COVID-19 [61]. These findings are consistent with studies in Filipino domestic workers and hospital clinicians, where fatigue was identified as the key symptom in the depression and anxiety network model [67, 68]. Thus, fatigue is possibly an influential symptom among workers across different contexts, which needs to be addressed to reduce depression, insomnia, and suicidality.

The bridge symptom ‘Motor Disturbance’ (PHQ8) found in our network model was also identified in previous depression and insomnia network analyses conducted in non-infected psychiatric practitioners [21], suggesting that motor disturbance is a prominent symptom linking depression and insomnia among MPHs in general. Following the end of China’s Dynamic Zero-COVID Policy, most MPHs worked long hours in hospitals due to a shortage of clinicians, which isolated them from their social support networks and limited their outdoor physical activities [69]. Moreover, “Motor Disturbance” (PHQ8) was directly associated with “Suicidality” (SU) in the flow network, suggesting that appropriate physical exercise could be a potential intervention in treating comorbid insomnia and depression, and reducing the risk of suicidality in this population [70].

There was a strong connection between “Guilt” (PHQ6) and “Sad mood” (PHQ2) with “Suicidality” (SU) in the flow network. Due to the awareness of their obligations, MHPs might feel guilty and sad if they were unable to perform well in their clinical practice following COVID-19 infection, which could increase the risk of suicidality [71]. Compared with insomnia symptoms, depressive symptoms were more strongly and directly associated with suicidality in the flow network, which is consistent with a consensus that depression is considered the most important predictor of suicide [14]. People who recovered from COVID-19 inflection might be at an increased risk for suicide, for instance, in a study of American young adults who were infected with COVID-19, 13.4% of the sample reported suicidal ideation, while 5.4% reported suicide plan and 1.3% reported suicide attempt [72]. Numerous studies have also suggested positive correlations between the severity of COVID-19 symptoms and an elevated risk of suicidal symptoms [14, 15, 72]. Thus, it is likely that high levels of post-COVID-19 depression might contribute to an increased risk of suicidality.

Strengths of this study included a large sample size of MHPs who recovered from COVID-19 and the use of network analyses. However, some limitations should be acknowledged. First, selection bias might exist as random sampling was not used. Second, response bias could not be eliminated since some MHPs, including those with severe insomnia or depression or those who were not interested in this survey, might not have responded. Third, this was a cross-sectional study, hence the causality between different symptoms could not be detected. Fourth, the previous status of COVID-19 infection was assessed through self-reported questions, which was not precise. Finally, the majority of the COVID-19 survivors had a mild infection (only 1.7% were hospitalized), thus the results would not be generalizable beyond non-hospitalized patients with COVID-19.

In conclusion, the study found that “Distress caused by sleep difficulties” (ISI7), “Interference with daytime functioning” (ISI5) and “Sleep dissatisfaction” (ISI4) were the key central symptoms in MHPs following their recovery from COVID-19. Additionally, “Suicidality” (SU) was closely associated with “Guilt” (PHQ6), “Sad Mood” (PHQ2), and “Motor Disturbances” (PHQ8). To reduce the negative outcomes caused by depressive and insomnia symptoms and suicidality, developing prevention and intervention strategies that target both central and bridge symptoms is important for MHPs following their recovery from COVID-19.

### Supplementary information

Supplemental materials

## Data Availability

The data of this study are available from the corresponding author upon reasonable request.
