# Supplementary material for: Prevalence and network structure of depression, insomnia and suicidality among mental health professionals who recovered from COVID-19: a national survey in China
Source: Transl Psychiatry. 2024 May 30;14:227. doi: 10.1038/s41398-024-02918-8 (PMC11139988; doi:10.1038/s41398-024-02918-8)
Supplement: Supplementary file 1 — Supplemental materials [file 41398_2024_2918_MOESM1_ESM.docx]

**Supplemental materials**

**Figure legends**

**Figure S1.** Bootstrapped confidence intervals of edge weights.

**Figure S2.** Bootstrapped test for confidence intervals of EI and bridge EI of depression and insomnia with suicidality network model

**Figure S3.** Bootstrapped difference test for edge weight of depression and insomnia with suicidality network model

**Figure S4.** Bootstrapped difference test for expected influence of depression and insomnia with suicidality network model

**Table S1.** Basic information and network inference of items used to assess depression, insomnia and suicidality (N=9,858)

| Nodes | Label | Recovered COVID-19 patients | | |
| --- | --- | --- | --- | --- |
|  |  | **M (SD)** | **EI** | **CCmarg*** |
| **Depressive symptoms** | | | | |
| Anhedonia | PHQ1 | 0.82 (0.869) | -0.40 | 0.576 |
| Sad Mood | PHQ2 | 0.58 (0.750) | 0.55 | 0.557 |
| Fatigue | PHQ4 | 0.94 (0.887) | 0.67 | 0.648 |
| Appetite | PHQ5 | 0.66 (0.839) | -1.19 | 0.539 |
| Guilt | PHQ6 | 0.45(0.724) | 0.35 | 0.666 |
| Concentration | PHQ7 | 0.54 (0.776) | -0.20 | 0.607 |
| Motor Disturbance | PHQ8 | 0.35 (0.664) | 0.21 | 0.735 |
| **Insomnia symptoms** | | | | |
| Difficulty falling asleep | ISI1 | 0.90 (0.904) | -0.02 | 0.616 |
| Difficulty staying asleep | ISI2 | 0.88 (0.923) | 0.40 | 0.59 |
| Problems waking up too early | ISI3 | 0.81 (0.903) | -0.95 | 0.558 |
| Sleep dissatisfaction | ISI4 | 1.56 (1.031) | 0.74 | 0.838 |
| Interference with daytime functioning | ISI5 | 0.74 (0.839) | 1.08 | 0.532 |
| Noticeability of sleep problems by others | ISI6 | 0.62 (0.811) | 0.04 | 0.544 |
| Distress caused by the sleep difficulties | ISI7 | 0.67 (0.856) | 1.34 | 0.524 |
| **Suicidality** | | | | |
|  |  | **N (%)** | **EI** | **CCmarg** |
| Any type of suicidality | SU | 771 (7.8) | -2.63 | 0.922 |
| Notes: SD: standard deviation; PHQ-9: Patient Health Questionnaire; ISI: Insomnia Severity Index; EI: Expected Influence (z-score); CCmarg: the proportion of correct classification normalized by the marginal distribution of the nodes; CCmarg*: the value after recoded to binary data | | | | |

**Table S2.** Weighted adjacency matrix of the depression and insomnia with suicidality network model

|  | PHQ1 | PHQ2 | PHQ4 | PHQ5 | PHQ6 | PHQ7 | PHQ8 | ISI1 | ISI2 | ISI3 | ISI4 | ISI5 | ISI6 | ISI7 |
| --- | --- | --- | --- | --- | --- | --- | --- | --- | --- | --- | --- | --- | --- | --- |
| PHQ1 |  |  |  |  |  |  |  |  |  |  |  |  |  |  |
| PHQ2 | 2.02 |  |  |  |  |  |  |  |  |  |  |  |  |  |
| PHQ4 | 1.59 | 1.07 |  |  |  |  |  |  |  |  |  |  |  |  |
| PHQ5 | 0.57 | 0.32 | 1.06 |  |  |  |  |  |  |  |  |  |  |  |
| PHQ6 | 0.06 | 1.78 | 0.66 | 0.60 |  |  |  |  |  |  |  |  |  |  |
| PHQ7 | 0.50 | 0.52 | 0.73 | 0.66 | 1.04 |  |  |  |  |  |  |  |  |  |
| PHQ8 | 0.54 | 0.36 | 0.31 | 0.77 | 1.23 | 1.84 |  |  |  |  |  |  |  |  |
| ISI1 | 0.00 | 0.00 | 0.47 | 0.36 | 0.00 | 0.04 | 0.00 |  |  |  |  |  |  |  |
| ISI2 | 0.09 | 0.08 | 0.06 | 0.10 | 0.00 | 0.21 | 0.00 | 1.62 |  |  |  |  |  |  |
| ISI3 | 0.00 | 0.00 | 0.15 | 0.08 | 0.00 | 0.00 | 0.30 | 0.33 | 1.52 |  |  |  |  |  |
| ISI4 | 0.18 | 0.00 | 0.61 | 0.00 | 0.00 | 0.12 | 0.00 | 1.37 | 1.55 | 1.39 |  |  |  |  |
| ISI5 | 0.31 | 0.00 | 0.53 | 0.06 | 0.12 | 0.27 | 0.00 | 0.80 | 0.39 | 0.36 | 1.36 |  |  |  |
| ISI6 | 0.00 | 0.00 | 0.00 | 0.06 | 0.08 | 0.08 | 0.56 | 0.69 | 0.58 | 0.49 | 0.00 | 1.96 |  |  |
| ISI7 | 0.00 | 0.38 | 0.17 | 0.10 | 0.19 | 0.15 | 0.40 | 0.73 | 0.82 | 0.45 | 0.92 | 1.82 | 1.90 |  |
| SU | 0.00 | 0.68 | 0.00 | 0.00 | 1.17 | 0.00 | 0.42 | 0.00 | 0.00 | 0.00 | 0.00 | 0.00 | 0.08 | 0.31 |

**
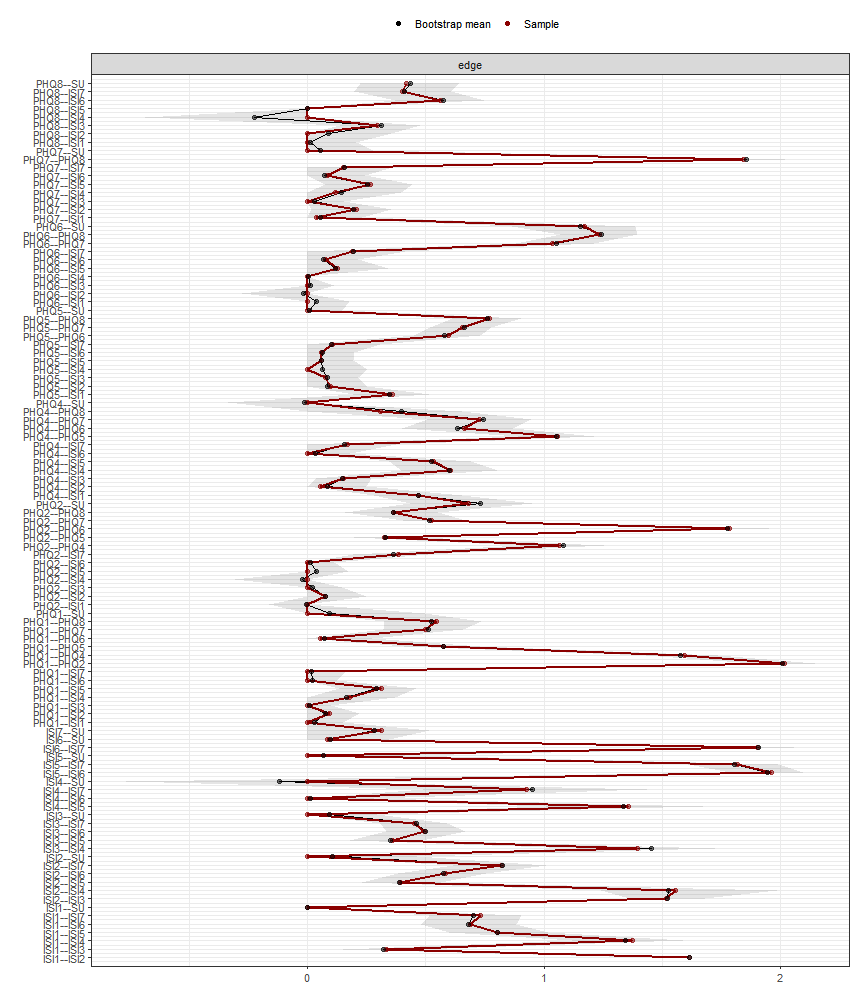
**

**Figure S1.** Bootstrapped confidence intervals of edge weights


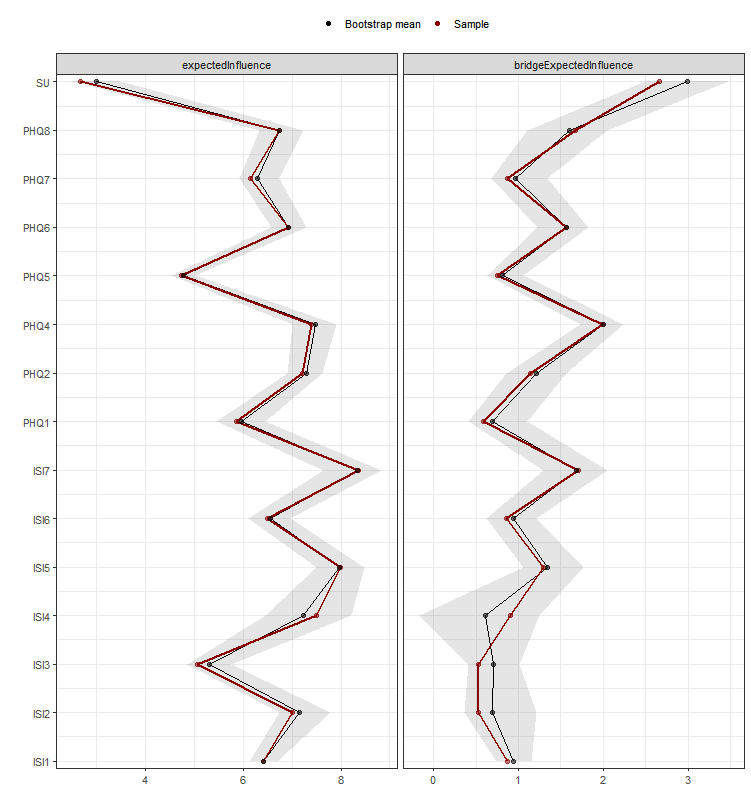


**Figure S2.** Bootstrapped test for confidence intervals of EI and bridge EI of depression and insomnia with suicidality network model

**
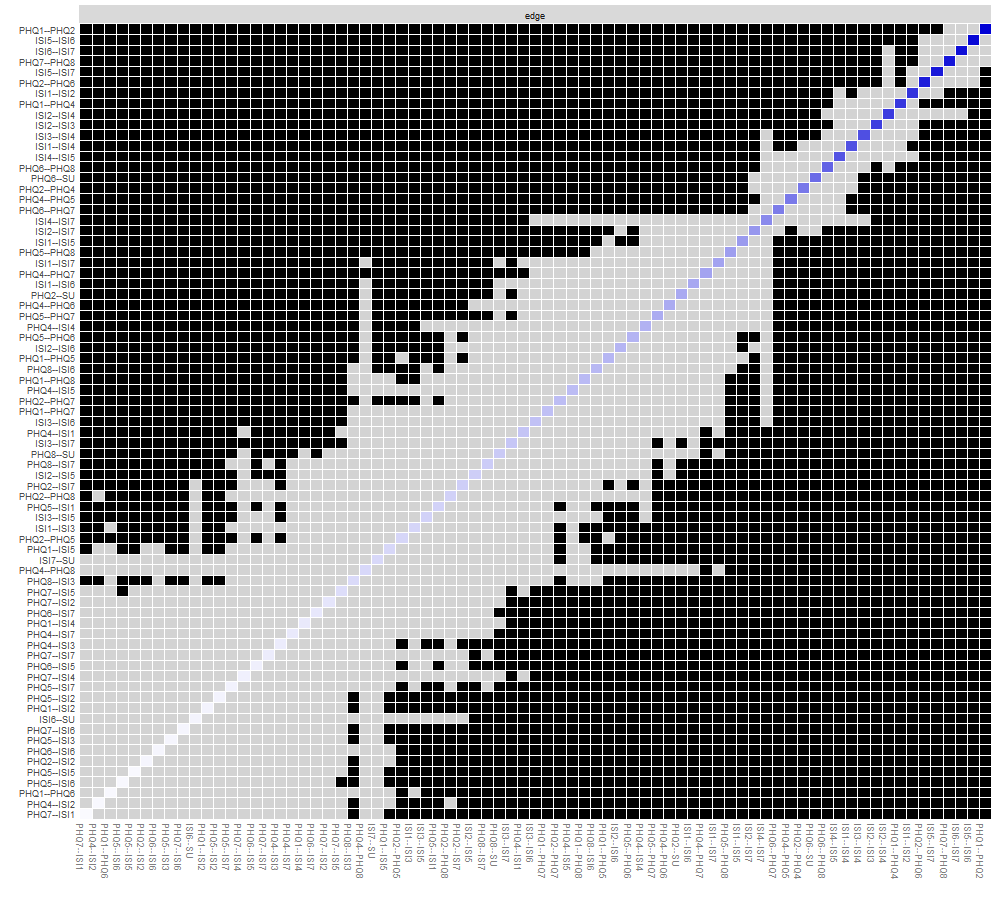
**

**Figure S3.** Bootstrapped difference test for edge weight of depression and insomnia with suicidality network model

**
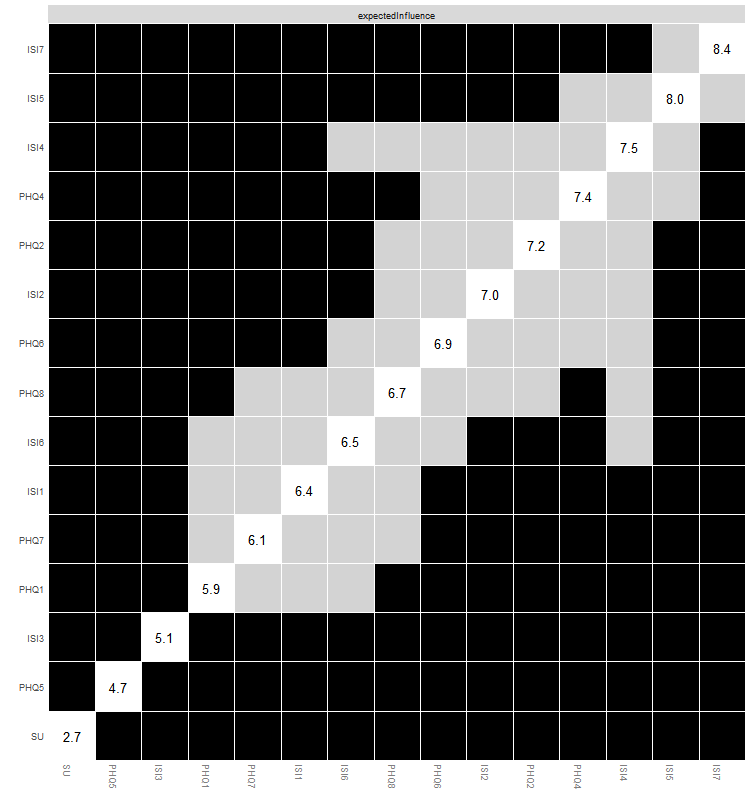
**

**Figure S4.** Bootstrapped difference test for expected influence of depression and insomnia with suicidality network model
